# Supplementary figures and images for: Analysis of the emergency response capacity of nursing staff for public health emergencies and the influencing factors: A cross-sectional study in China
Source: PLoS One. 2025 May 30;20(5):e0323992. doi: 10.1371/journal.pone.0323992 (PMC12124578; doi:10.1371/journal.pone.0323992)

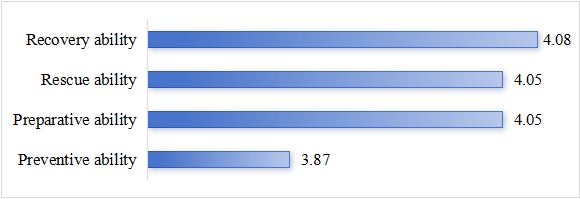

Supplement: S2 Fig — (TIF) [file pone.0323992.s002.tif]
